# Paraneoplastic Syndromes Mimicking Dermatomyositis and Remitting Seronegative Symmetrical Synovitis With Pitting Edema (RS3PE) Syndrome in Cancer of Unknown Primary Origin: A Case Report

**DOI:** 10.7759/cureus.81376

**Published:** 2025-03-28

**Authors:** Shoji Mochizuki, Ryuichi Ohta, Yuki Konya, Kosuke Isomoto, Takayuki Takahama, Junko Tanizaki, Hidetoshi Hayashi

**Affiliations:** 1 Department of Medical Oncology, Kindai University Faculty of Medicine, Sayama, JPN; 2 Department of Community Care, Unnan City Hospital, Unnan, JPN

**Keywords:** arthritis, carcinoma, dermatomyositis, neoplasms, paraneoplastic syndromes, remitting seronegative symmetrical synovitis with pitting edema syndrome, rheumatoid, squamous cell, steroids, unknown primary

## Abstract

A 70-year-old male patient presented with fever, polyarthritis, systemic muscle weakness and pain, and skin rash, initially suspected to be an autoimmune disorder. Imaging revealed right supraclavicular and paratracheal lymphadenopathy, and a right supraclavicular lymph node biopsy confirmed squamous cell carcinoma. Still, the primary site remained unidentified, leading to a diagnosis of cancer of unknown primary origin (CUP). Laboratory tests showed no positive autoantibodies such as anti-Jo-1, anti-ribonucleoprotein (RNP), anti-Smith (Sm), and anti-SS-A antibodies, and a skin biopsy of the back indicated panniculitis with neutrophilic infiltration. Given the absence of infectious or autoimmune causes, the symptoms were attributed to paraneoplastic syndrome (PNS) associated with CUP, mimicking dermatomyositis and remitting seronegative symmetrical synovitis with pitting edema (RS3PE) syndrome. Treatment with prednisolone (15 mg/day) led to the rapid resolution of joint pain, rash, and fever. Chemotherapy with carboplatin and paclitaxel for CUP was initiated with minimal adverse effects, allowing for continued outpatient management. This case highlights the importance of considering PNS when collagen disease-like symptoms are present in malignancy, particularly in CUP. Early recognition and corticosteroid therapy can improve performance status, enabling timely cancer treatment. Identifying atypical PNS presentations in CUP remains challenging, but a multidisciplinary approach can aid in diagnosis and management.

## Introduction

Malignancies can cause systemic symptoms through various immune mechanisms, including collagen disease-like symptoms such as arthritis, bursitis, skin rashes, and Raynaud's phenomenon [1]. These symptoms are considered part of paraneoplastic syndrome (PNS), which occurs in approximately 5% of all PNS cases and is relatively rare [2]. Representative conditions include polymyositis/dermatomyositis, which coexists in about 10% to 20% of lung, ovarian, gastric, and pancreatic cancers [3]. Rheumatoid factor-positive arthritis can be associated with lung and breast cancers [4]. Systemic sclerosis-like symptoms may be seen in lung and breast cancers [5]. Sjögren's syndrome-like symptoms are associated with lymphomas [6]. However, the frequency and presentation of PNS with collagen disease-like symptoms in cases of cancer of unknown primary origin (CUP) remain variable and unclear.

Here, we report a case of a patient who presented with fever, generalized joint pain, and diverse skin findings and was ultimately diagnosed with PNS secondary to CUP. Although the patient's physical findings suggested dermatomyositis, the serological results did not support this diagnosis. In most cases, malignancies are identified through cancer screening initiated after a diagnosis of dermatomyositis. This case highlights that CUP alone can present with dermatomyositis-like symptoms. We discuss how medical oncologists should approach and manage such presentations.

## Case presentation

**Figure 1:**
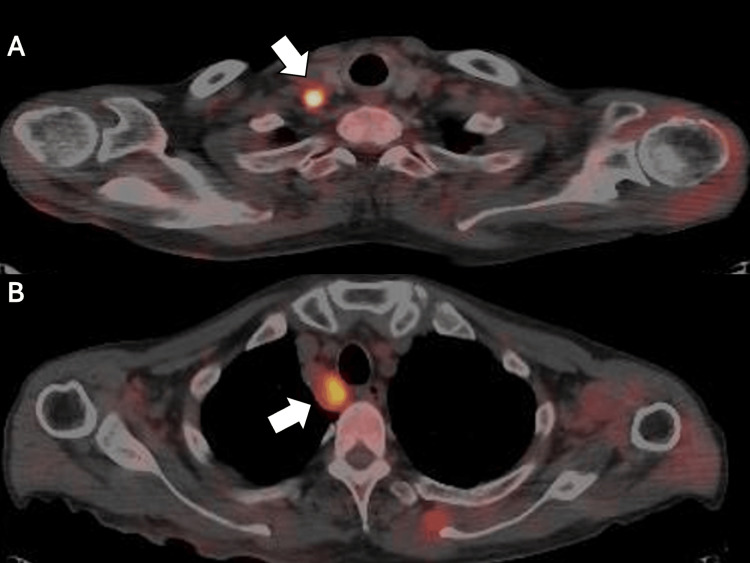
Positron emission tomography-computed tomography demonstrating the uptake in the right supraclavicular (A) and right paratracheal (B) lymph nodes (white arrows).

A 70-year-old male patient was referred to our hospital for evaluation of fever, muscle weakness and pain, joint pain, edema in both hands and feet, rash on the anterior chest, and investigation of CUP. Four months before presentation, he developed bilateral lower leg edema and generalized fatigue. He visited a primary care physician and was referred to a regional general hospital three months prior for further investigation. Laboratory tests revealed elevated C-reactive protein (CRP), hypoalbuminemia, and anemia. Comprehensive evaluations, including upper and lower gastrointestinal endoscopy, showed no apparent malignant findings, according to European Society for Medical Oncology (ESMO) guidelines for CUP [7]. Positron emission tomography-computed tomography (PET-CT) demonstrated uptake in the right supraclavicular and paratracheal lymph nodes (Figure 1).

**Figure 2:**
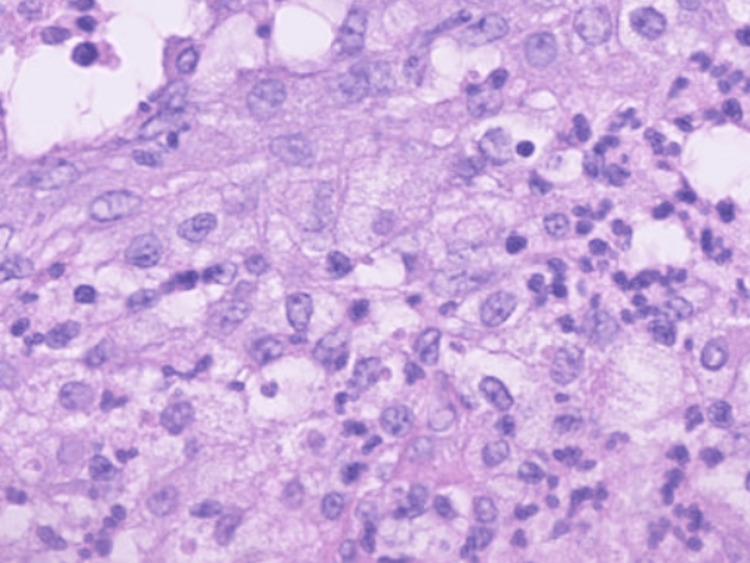
A right supraclavicular lymph node biopsy showing squamous cell carcinoma with keratinization and intercellular bridges, supporting the diagnosis of metastatic squamous cell carcinoma.

A right supraclavicular lymph node biopsy suggested squamous cell carcinoma with keratinization and intercellular bridges, supporting the diagnosis of metastatic squamous cell carcinoma. Immunohistochemical staining showed positivity for p40 and CK5/6, confirming the diagnosis (Figure 2).

The patient was referred to our hospital for further evaluation and treatment of CUP. He did not have night sweats, shortness of breath, cough, abdominal pain, or diarrhea. The patient's social history included smoking 20 cigarettes per day for 40 years and occasional alcohol consumption. His past medical history included hypertension and hyperuricemia. His medications at the time of presentation included urarisid (100 mg), febuxostat (30mg), ambroxol (45mg), and rupatadine (10mg) daily.

**Figure 3:**
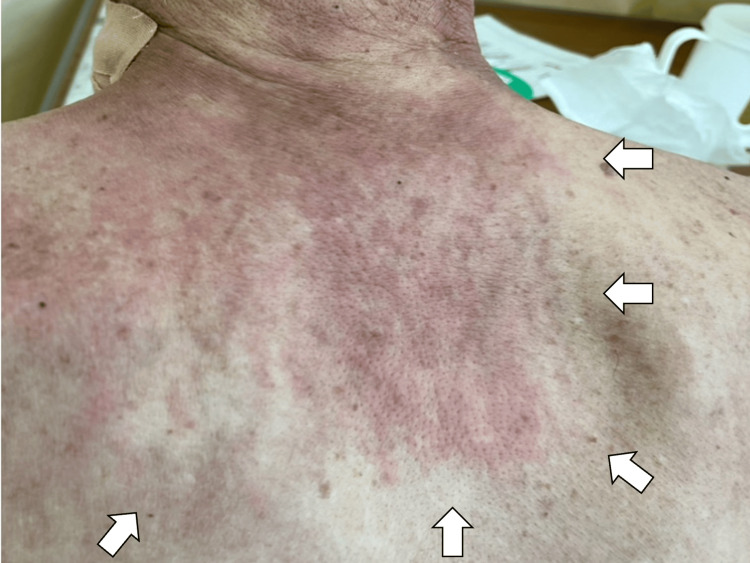
Excoriated-like rashes on the back (white arrows)

Upon arrival, his vital signs were as follows: consciousness was clear, blood pressure 134/56 mmHg, heart rate 87 beats per minute, respiratory rate 16 breaths per minute, body temperature 37.4°C, and oxygen saturation 96% on room air. His Eastern Cooperative Oncology Group (ECOG) performance status (PS) was two. Physical examination revealed excoriated-like rashes on the chest and back, suggesting shawl sign of dermatomyositis (Figure 3).

The wrists, ankles, and feet exhibited erythema, warmth, swelling, pitting edema, and tenderness. The physical finding showed tenderness on the bilateral shoulders and thighs. The laboratory findings showed leukocytosis and elevated CRP levels. Matrix metalloproteinase-3 (MMP-3) was elevated, whereas creatine kinase (CK) and lactate dehydrogenase (LDH) levels were within normal limits (Table 1).

**Table 1:** Initial laboratory data of the patient

Parameter	Level	Reference range
White blood cells	9.72	3.5–9.1 × 10^3^/μL
Neutrophils	86.7	44.0–72.0%
Lymphocytes	5.7	18.0–59.0%
Hemoglobin	8.5	11.3–15.2 g/dL
Hematocrit	27.9	33.4–44.9%
Mean corpuscular volume	85.8	79.0–100.0 fl
Platelets	42.6	13.0–36.9 × 10^4^/μL
Total protein	6.9	6.5–8.3 g/dL
Albumin	2.6	3.8–5.3 g/dL
Aspartate aminotransferase	13	8–38 IU/L
Alanine aminotransferase	10	4–43 IU/L
Lactate dehydrogenase	140	121–245 U/L
Blood urea nitrogen	17	8–20 mg/dL
Creatinine	0.93	0.40–1.10 mg/dL
Serum sodium (Na)	136	135–150 mEq/L
Serum pottassium (K)	4.3	3.5–5.3 mEq/L
Serum chloride (Cl)	105	98–110 mEq/L
C-reactive protein	9.333	<0.30 mg/dL
Immunoglobulin G (IgG)	1511	870–1700 mg/dL
Immunoglobulin M (IgM)	52	35–220 mg/dL
Immunoglobulin A (IgA)	226	110–410 mg/dL
Matrix metalloproteinase-3 (MMP-3)	208	36.9－121ng/ml
Leukocyte	Negative	Negative
Protein	Negative	Negative
Blood	Negative	Negative

Antinuclear antibodies were negative, and tests for anti-Jo-1, anti-ribonucleoprotein (RNP), anti-Smith (Sm), and anti-SS-A antibodies were also negative.

**Figure 4:**
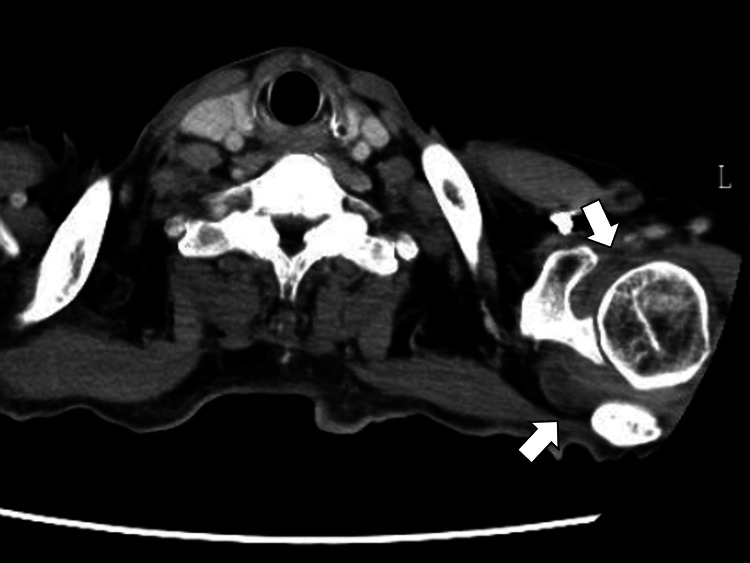
Contrast-enhanced computed tomography showing fluid accumulation in the left acromioclavicular joint (white arrows)

At the time of admission, he had a persistent fever, tenderness in both hands and feet, and rashes on the anterior chest and back. Blood cultures and contrast-enhanced computed tomography (CT) from the neck to the pelvis were performed to evaluate for sepsis and abscess formation. Blood cultures were negative. Contrast-enhanced CT showed fluid accumulation in the left acromioclavicular joint, left axilla, right back, and right upper mediastinal lymphadenopathy (Figure 4).

The left shoulder joint bursa was aspirated, and the aspirate was sent for culture. The culture revealed only inflammatory findings without bacterial growth or malignant cells.

**Figure 5:**
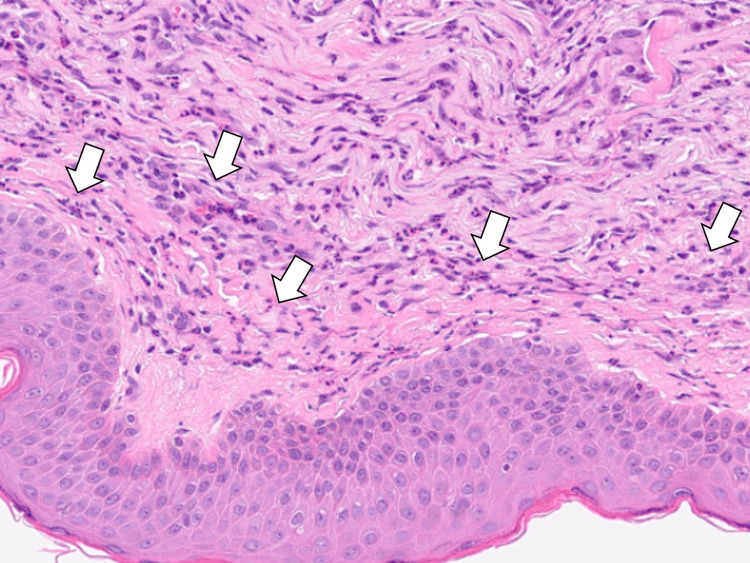
A skin biopsy revealing lobular and septal panniculitis with perivascular lymphocytic infiltration and neutrophilic predominance (white arrows)

Given the possibility of autoimmune or dermatological diseases in addition to cancer of unknown primary origin, consultations were made with the dermatology and rheumatology departments. The dermatology team performed a back skin biopsy, and histopathological examination revealed lobular and septal panniculitis with perivascular lymphocytic infiltration and neutrophilic predominance, supporting a paraneoplastic process (Figure 5).

The rheumatology team noted the absence of specific autoantibodies and determined that the symptoms were consistent with a PNS associated with cancer of unknown primary origin.

The patient was clinically diagnosed with PNS presenting as dermatomyositis-like and remitting seronegative symmetrical synovitis with pitting edema (RS3PE) syndrome-like symptoms secondary to cancer of unknown primary origin. On day 14 of hospitalization, prednisolone 15 mg/day was initiated for PNS treatment. By day 16, joint tenderness had improved, and his body temperature normalized to approximately 37°C, with the resolution of the chest and back rashes. The patient's ECOG PS improved from two to one.

Following a stable clinical course, chemotherapy was initiated on day 23 for cancer of unknown primary origin with carboplatin (area under the curve (AUC) five) and paclitaxel (160 mg/m²) in a three-week cycle. The only observed adverse effect was Grade 1 constipation. The patient was otherwise stable and was discharged on the 27^th^ day of admission. In the outpatient department, he was continually treated with the same chemotherapy every three weeks.

## Discussion

This case shows that dermatomyositis and RS3PE syndrome-like symptoms can present simultaneously as PNSs associated with CUP. By appropriately differentiating these conditions and excluding infections, concurrent use of steroids alongside cancer treatment could effectively control the symptoms.

The possibility of simultaneous presentation of dermatomyositis and RS3PE syndrome-like symptoms as PNS in CUP is an essential consideration for future cases. Paraneoplastic syndromes are organ dysfunctions occurring at sites distant from the primary tumor or its metastases, observed in approximately 5% to 10% of cancer patients, with some presenting collagen disease-like symptoms [8]. Compared to conventional collagen diseases, PNS often presents atypical symptoms and clinical courses, with poor responses to standard treatments [1,9,10]. In this case, however, symptoms rapidly improved after initiating prednisolone, suggesting that the resistance to steroid treatments might not be a definitive criterion for distinguishing PNS. While PNS has been reported to be less responsive to corticosteroids, the degree of responsiveness may vary depending on factors such as the extent of immune activation triggered by the malignancy, differences in cytokine-mediated inflammation, and the interplay between tumor progression and autoimmunity. In particular, malignancy-associated inflammation can modulate immune tolerance and alter steroid sensitivity, leading to variable clinical outcomes [11,12]. These factors highlight the complexity of PNS pathophysiology and underscore the need for a nuanced approach in distinguishing PNS from autoimmune diseases. Given this complexity, clinicians should not rely solely on corticosteroid responsiveness to differentiate PNS from other autoimmune diseases.

Furthermore, in cases where a single autoimmune disease does not explain the clinical presentation, it is necessary to consider PNS actively. Collagen disease-like symptoms related to PNS can clinically resemble relapsing polychondritis, hypertrophic osteoarthropathy, polymyalgia rheumatica, RS3PE syndrome, and dermatomyositis [13,14]. The concurrent presentation of multiple such symptoms, as seen in this case with dermatomyositis and RS3PE syndrome, is uncommon in standard collagen disease practice [15]. Therefore, considering the possibility of PNS and conducting thorough investigations is essential when such overlapping symptoms are observed.

The response of dermatomyositis and RS3PE syndrome-like symptoms to steroids in PNS cases appears favorable, emphasizing the need for the timely use of prednisolone in clinical practice. The RS3PE syndrome typically affects elderly males and is a form of seronegative arthritis seen predominantly in those over 60 years old, especially in their 70s, with an acute onset [16]. It is characterized by symmetric arthritis with pitting edema in the hands and feet, negative autoantibodies, the absence of bone erosion, and a marked response to low-dose corticosteroids with minimal relapse [16]. The underlying pathophysiology is believed to involve heightened immunogenicity due to epithelial tumor infiltration or the tumor microenvironment [16]. Chronic B-cell activation mediated by tumor necrosis factor (TNF) superfamily members such as B-cell activating factor (BAFF) and reduced apoptosis activity have also been implicated [16]. Occasionally, dementia symptoms also develop in the process of RS3PE syndrome, impinging on the performance status of patients and starting chemotherapy when complicated with cancers [17].

A similar pathophysiological mechanism may underlie dermatomyositis in PNS cases, contributing to favorable steroid responsiveness and symptom improvement with corticosteroid use [3,18]. In this case, the early initiation of prednisolone improved the patient’s PS, allowing for the timely introduction of chemotherapy for CUP. Delays in treating collagen disease-like symptoms associated with PNS can occur due to differences in understanding between medical specialties. Therefore, in cases of collagen disease-like symptoms in CUP, the use of prednisolone as a diagnostic treatment can be a valuable practice for medical oncologists to ensure the smooth management of chemotherapy.

## Conclusions

This case suggests the possibility of dermatomyositis and RS3PE-like symptoms coexisting as paraneoplastic syndromes in CUP. When multiple collagen disease-like symptoms are observed, it is crucial to consider the possibility of CUP and conduct further investigations. Early treatment of PNS symptoms can lead to improvement in PS, facilitating earlier initiation of chemotherapy and enabling smoother cancer management for patients.
